# Do we still need breast cancer screening in the era of targeted therapies and precision medicine?

**DOI:** 10.1186/s13244-020-00905-3

**Published:** 2020-09-25

**Authors:** Rubina Manuela Trimboli, Paolo Giorgi Rossi, Nicolò Matteo Luca Battisti, Andrea Cozzi, Veronica Magni, Moreno Zanardo, Francesco Sardanelli

**Affiliations:** 1 Department of Biomedical Sciences for Health, Università degli Studi di Milano, Via Mangiagalli 31, 20133 Milan, Italy; 2 Epidemiology Unit, Azienda USL–IRCCS di Reggio Emilia, Via Amendola 2, 42122 Reggio Emilia, Italy; 3 Breast Unit–Department of Medicine, The Royal Marsden NHS Foundation Trust, Downs Road, Sutton, London, SM2 5PT UK; 4 Breast Cancer Research Division, The Institute of Cancer Research, 15 Cotswold Road, Sutton, London, SM2 5NG UK; 5 Medical School, Università degli Studi di Milano, Via Festa del Perdono 7, 20122 Milan, Italy; 6 Unit of Radiology, IRCCS Policlinico San Donato, Via Morandi 30, 20097 San Donato Milanese, Italy

**Keywords:** Breast neoplasms, Mammography, Cancer screening, Prognosis, Precision medicine

## Abstract

Breast cancer (BC) is the most common female cancer and the second cause of death among women worldwide. The 5-year relative survival rate recently improved up to 90% due to increased population coverage and women’s attendance to organised mammography screening as well as to advances in therapies, especially systemic treatments. Screening attendance is associated with a mortality reduction of at least 30% and a 40% lower risk of advanced disease. The stage at diagnosis remains the strongest predictor of recurrences. Systemic treatments evolved dramatically over the last 20 years: aromatase inhibitors improved the treatment of early-stage luminal BC; targeted monoclonal antibodies changed the natural history of anti-human epidermal growth factor receptor 2-positive (HER2) disease; immunotherapy is currently investigated in patients with triple-negative BC; gene expression profiling is now used with the aim of personalising systemic treatments. In the era of precision medicine, it is a challenging task to define the relative contribution of early diagnosis by screening mammography and systemic treatments in determining BC survival. Estimated contributions before 2000 were 46% for screening and 54% for treatment advances and after 2000, 37% and 63%, respectively. A model showed that the 10-year recurrence rate would be 30% and 25% using respectively chemotherapy or novel treatments in the absence of screening, but would drop to 19% and 15% respectively if associated with mammography screening. Early detection *per se* has not a curative intent and systemic treatment has limited benefit on advanced stages. Both screening mammography and systemic therapies continue to positively contribute to BC prognosis.

## Key points

- The stage at diagnosis is still crucial in determining survival outcomes for breast cancer.
- Screening attendance is associated with a reduction of advanced-stage disease.
- Novel endocrine and anti-human epidermal growth factor receptor 2 (HER2)-targeted therapies have substantially improved survival.
- Early diagnosis and personalised treatments synergistically contribute to improve prognosis.
- We do still need breast cancer screening in the era of precision medicine.

## Introduction

Breast cancer is the most common female cancer worldwide, accounting for 30% of all new cancer diagnoses in women [1]. Social and economic trends are associated with a continuous increase in incidence rates by approximately 0.5% per year [1, 2]. Ageing population, maternity delay and low parity, obesity and sedentary lifestyle – along with an escalation in the diffusion of breast cancer mammography screening – contributed to this increase in high-income countries [2].

Traditionally, survival outcomes are influenced by tumour size, nodal involvement, grade, hormone receptor (HR) and human epidermal growth factor receptor 2 (HER2) status. Although breast cancer remains the second leading cause of death among women after lung cancer, the 5-year relative survival rate in the United States has improved from 79% in 1984–1986 to 91% in 2008–2014 [3, 4]. Similar trends have been observed in Europe: in Italy, survival improved from 80% in 1995 to 88% in 2010 [5].

**Fig. 1:**
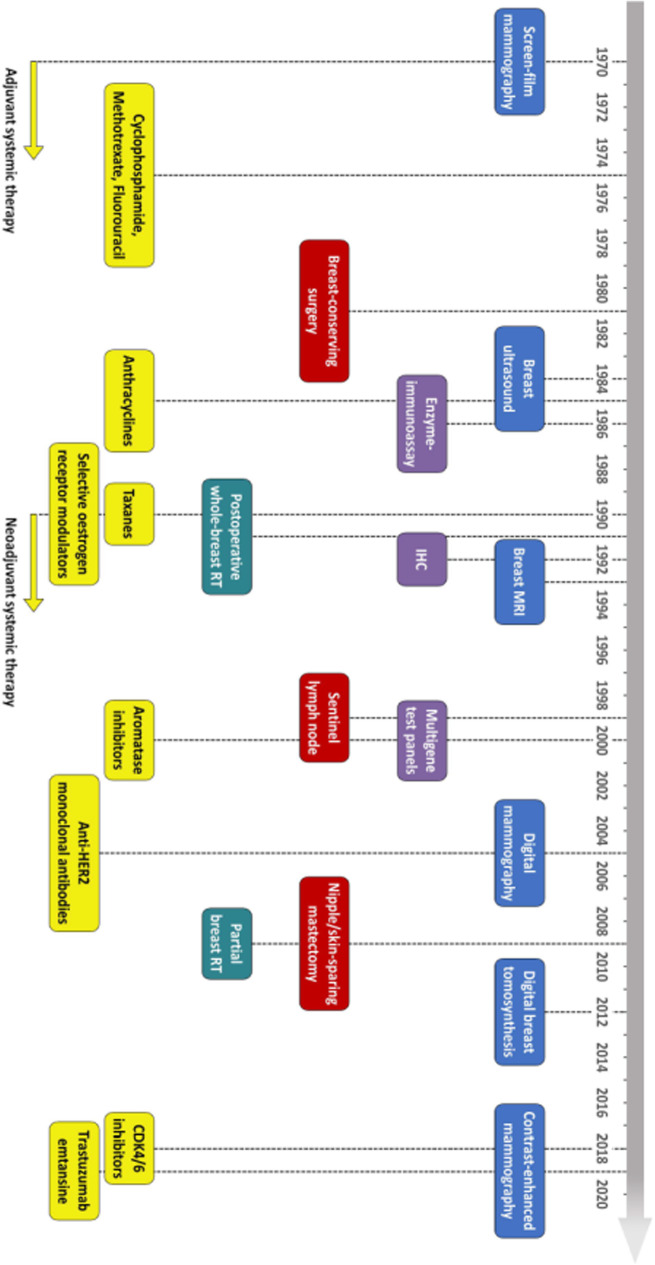
Key advances in the recent history of breast cancer care (the date of introduction of each innovation was based on literature review with reference to first large studies confirming its clinical value). Specific evolutions or improvements are colour-coded. Imaging is in light blue, pathology in violet, surgery in red, radiation therapy in blue-green, systemic treatments in yellow. RT, radiation therapy: IHC, immunohistochemistry; MRI, magnetic resonance imaging; CDK4/6, cyclin-dependent kinase 4/6

As detailed by the timeline in Fig. 1, breast cancer care has substantially evolved over the past fifty years, with non-negligible changes in screening and diagnostics, histological analysis, surgery, radiation therapy, and systemic treatments. The implementation and diffusion of screening mammography and various improvements in systemic anticancer treatments have been the main drivers of these changes [6]. In Europe, 63.9 million women had access to population-based breast cancer screening in 2016 compared with 54.4 million in 2007, with 88% of the estimated target population completing rollout, compared with 41% in 2007. Nevertheless, a wide geographical variability in invitation coverage still exists [7]. Similarly, in the 2000s, the use of chemotherapy has increased up to 80%, and the use of tamoxifen up to 50% for patients with oestrogen receptor-positive tumours: nowadays, in Italy, about 50% of patients undergoing surgery for stage I–III breast cancer receive systemic treatments that include targeted therapy [8], while in the United States targeted therapy is administered to almost 20% of patients with stage I–II disease and to over 60% of those with stage III disease [9]. Of note, along with traditional prognostic factors, gene expression profiling is now increasingly adopted, aiming to personalise therapeutic approaches and escalate or de-escalate systemic treatments [10]. In this complex framework, an evaluation of the impact of screening and systemic treatments on breast cancer prognosis looks challenging: their relative contribution may substantially differ across countries with different screening attendance rate and access to anticancer treatments.

Benefits deriving from screening and early diagnosis may become questionable when considering the efficacy of current systemic treatment options. In a theoretical model, a break-even point can be hypothesised when the advantages of early diagnosis by screening are nullified by the efficacy of individualised therapies. Nonetheless, detecting breast cancer before it is no longer curable should still be considered an advantage for the patient, since it remains unlikely that currently available therapies could be so effective without an early diagnosis. Any modification to this balance relies on the potential of systemic treatments to kill cancer cells at any stage, reducing or nullifying the screening benefit.

This critical review highlights the major landmark improvements in mammography screening and systemic treatment, to appraise their impact on breast cancer prognosis and to question the role of breast cancer screening in the era of precision medicine.

### Mammography screening

Mammography screening was introduced in 1956 for the “detection of early cancer of the breast” [11]. In the 1970s, several randomised controlled trials confirmed a beneficial improvement of mammography screening on breast cancer mortality [12]. Such benefit pertains to women aged 50 to 74 years and is maximal, about 30%, in those aged 60 to 69 years [13–16]. Mammography screening programs have the general aim of reducing breast cancer-specific mortality both by minimising the risk of diagnosing breast cancer in the advanced stage and by maximising the efficacy and safety of anticancer treatments and their impact on prognosis.

The first x-ray units dedicated to mammography used x-ray film and paired fluorescent screens to capture the image, in the so-called screen-film mammography, which has been gradually replaced since the early 2000s by digital mammography units. In 2005, a landmark study by Pisano et al. [17] compared the diagnostic accuracy of digital and screen-film mammography in 42,760 asymptomatic women. At receiver operating characteristic analysis over the entire population, the areas under the curve (AUCs) of the two methods differed by a non-significant 0.03. However, the digital technique was significantly superior among women over 50 (0.15 AUC difference), among women having heterogeneously or extremely dense breasts (0.11 AUC difference), and among women being in pre- or perimenopause (0.15 AUC difference). Digital mammography allowed also to deliver a lower radiation dose and resulted in easier image storage and subsequent access. Its initially higher costs dwindled quite quickly, its uptake steadily increased [18, 19]. By 2015, 96% of all mammography units in the United States had gone digital [20].

The incidence of advanced-stage tumours in the target population is a specific proxy indicator of screening efficacy, given the absence of confounding effects of treatments at the time of diagnosis. However, studies on this topic have adopted different thresholds in defining the severity of the diseases and are characterised by poor to fair overall quality. Nevertheless, a meta-analysis of trials combined their results – including the most severe disease categories available – and showed a significant reduction in the risk of advanced-stage disease for women aged 50 years or older (relative risk 0.62, 95% confidence interval [CI] 0.46–0.83) randomly assigned to undergo screening: this benefit was however not seen in women aged 39 to 49 years [14].

More recently, a prospective Italian cohort study was conducted on 413,447 women undergoing screening in the 1990s and followed up for 13 years as part of the IMPACT project [21]. Screening attendance was positively associated with: (a) a 39% reduction of the incidence of pT2–T4 lesions (66.3‰ versus 108.6‰, incidence rate ratio 0.61, 95% CI 0.57–0.66), including a 28% reduction of pT2 lesions and a 68% reduction of pT3–T4 lesions; (b) a 28% reduction in the incidence of stage II–IV disease (130.1‰ versus 180.6‰, incidence rate ratio 0.72, 95% CI 0.68–0.76), including a 35% reduction of stage IIB, a 43% reduction of stage III, and a 73% reduction of stage IV. Notably, patients undergoing screening also benefited from a 17% reduction in the incidence of poorly differentiated carcinomas and from a 50% increase of breast-conserving surgery rates. These results were also confirmed following adjustments to exclude self-selection bias, a significant reduction in the use of mastectomy being also observed [22].

Another interesting large population study – including 549,091 women across nine Swedish counties [23] – was conducted to evaluate the impact of screening mammography on breast cancer mortality. The study used an analytic strategy focusing on the incidence of fatal breast cancers within 10 years from the date of diagnosis, whereas other studies had been retroactively considering 10 years from the date of death. Women who attended mammography screening had a significant 41% reduction in their risk of dying of breast cancer within 10 years (relative risk 0.59, 95% CI 0.51–0.68) and a 25% reduction in the rate of advanced breast cancer (relative risk 0.75, 95% CI 0.66–0.84), regardless of the recent improvements in systemic treatments.

In 2019, the American Cancer Society and the National Cancer Institute published cancer treatment and survivorship statistics in the United States. Most patients (44%) were diagnosed with stage I disease, 30% with stage II, 9% with stage III and 5% with stage IV. The 5-year breast cancer relative survival ranged from approximately 100% for stage I disease to 26% for stage IV breast cancer [9]. Interestingly, in 2018 Mariotto et al. [24] provided the first population-based summaries of the risk of breast cancer recurrence in United States women, using cancer registry disease-specific survival: stage remained the strongest predictor of the risk of recurrence, along with age (60–74 years) and HR-negative status. Thus, we can still affirm that tumour stage has a substantial impact on prognosis and on the risk of disease recurrence.

### Systemic treatments

Chemotherapy has been used in the adjuvant setting since the early 1970s when landmark studies in the United States and in Italy documented a benefit of regimens such as L-phenylalanine mustard or the combination of cyclophosphamide, methotrexate and 5-fluorouracil (CMF) in patients with node-positive breast cancer [25, 26]. In the 1980s and 1990s, anthracyclines and taxanes proved to be more effective than CMF [27]; in the meantime, tamoxifen was found to substantially improve the survival of women with HR-positive tumours [28], and later on, aromatase inhibitors (AIs) proved to further improve outcomes in postmenopausal patients. During the 2000s, anti-HER2 therapies were developed as one of the first targeted systemic treatment options, changing dramatically the management and prognosis of HER2-positive breast cancer; in the same decade, genome expression profiling was deployed in routine practice, further improving the personalisation of breast cancer treatments [29]. Nonetheless, patient selection remains crucial to maximise efficacy and safety of systemic treatments. The current standard of care for different disease subtypes is summarised below.

#### Luminal breast cancer

The introduction of AIs has been a key improvement in the management of HR-positive, HER2-negative breast cancer [9, 30]. Nowadays, AIs are standard of care for postmenopausal women, based on several studies that have documented better recurrence-free survival and disease-specific mortality in women treated with AIs compared with tamoxifen [19]. Two large phase III studies (the SOFT and TEXT studies) confirmed the role of AIs also for premenopausal patients and/or with high-risk disease, along with ovarian function suppression justified by a recurrence-free survival benefit [31]. An extended course of adjuvant endocrine therapy may also further improve outcomes, especially following upfront use of tamoxifen [32–39], since late recurrences remain a relevant issue for patients with luminal breast cancer [40]. Cyclin-dependent kinase 4/6 inhibitors are also being investigated in the adjuvant setting in several clinical trials [41–44], which might in due course change the current treatment paradigm in this setting.

#### HER2-positive breast cancer

Targeted agents are the foundation of precision medicine, which involves the use of drugs interfering with specific molecular alterations that drive tumour growth and spread: for example, targeted anti-HER2 agents have radically changed the natural history and prognosis of HER2-positive disease. The use of the anti-HER2 monoclonal antibody trastuzumab for 1 year, along with chemotherapy, is now standard of care for tumours with a size of 5 mm or greater and for tumours of any size with nodal involvement, based on the substantial disease-free and overall survival benefits that have been reported when compared with the use of chemotherapy alone (disease-free survival, hazard ratio for recurrence 0.60, 95% CI 0.50–0.71; overall survival, hazard ratio for mortality 0.66, 95% CI 0.57–0.77) [45]. Moreover, additional benefits have been documented with adjuvant treatment escalation in higher-risk tumours: adding novel anti-HER2 agents, such as pertuzumab and neratinib, improves disease-free survival in women with node-positive disease [46] and recurrence rates in patients with large and HR-positive, HER2-positive tumours [47]. On the other hand, adjuvant treatment de-escalation has been investigated for patients with a lower risk of breast cancer recurrence in order to minimise the impact of systemic treatment on safety and quality of life [48]. Response to preoperative systemic treatment is a prognostic factor in patients with HER2-positive breast cancer, with better survival outcomes in patients without invasive disease at the surgical specimen, defined as ypT0/Tis ypN0 [49]. Therefore, neoadjuvant systemic treatment remains a very reasonable approach [50] and the addition of pertuzumab to trastuzumab to chemotherapy has become a standard of care, considering the improvements in pathological complete response rates [51, 52].

#### Triple-negative breast cancer

The systemic treatment of HR-negative and HER2-negative breast cancer is still largely limited to the use of chemotherapy. Adjuvant chemotherapy remains standard of care for patients with triple-negative breast cancers, either of 5 mm or greater or with pathologically involved lymph nodes [53]. In this specific setting, several trials are currently investigating the role of novel agents such as immunotherapy, which aims to boost the immune response against the tumour [54–56]. Neoadjuvant chemotherapy is the preferable approach in patients with locally advanced disease or in those who are not candidates for upfront surgery. Nonetheless, the role of neoadjuvant systemic therapy has also expanded, aiming to improve surgical operability and cosmetic outcomes but also to test the chemosensitivity of breast cancer in vivo, which has relevant prognostic implications [49, 57].

#### Gene expression profiling

Genomics and the ability to evaluate simultaneously the expression of multiple genes led to the development of gene expression profiles, which have been validated to identify patients with a higher risk of disease recurrence who may benefit from the use of adjuvant chemotherapy. Oncotype Dx has been validated both as a prognostic and a predictive tool, although Mammaprint [58], EndoPredict [59, 60], Breast Cancer Index [61] and Predictor Analysis of Microarray 50 (PAM50) [62, 63] may also be used. Oncotype Dx identifies women with node-negative, HR-positive breast cancer whose prognosis is so favourable that the absolute benefit of chemotherapy is likely to be very low. Patients with HR-positive node-negative cancers derive substantial benefit from chemotherapy when their score is high, typically higher than 25. On the other hand, if their score is low or midrange (lower than 25), adding chemotherapy to endocrine treatment for women over 50 showed no benefit, although younger women may experience some benefit [64, 65]. While the use of Oncotype Dx in patients with node-positive, HR-positive breast cancer is supported by less robust evidence, it has been considered [66] and is currently being investigated [67].

### The winning weapon: mammography screening plus systemic treatments

Defining the relative contribution of screening mammography and systemic treatments to improve breast cancer outcomes is a challenging task. The increasing use of screening and the gradual implementation of more effective therapeutic approaches occurred over nearly the same period since the 1970s. In the meantime, breast surgery has also evolved, along with the introduction of sentinel lymph node biopsy and radiation therapy. Overall, each of all these developments substantially contributed to improve patient outcome.

The Cancer Intervention and Surveillance Modelling Network used modelling techniques to provide estimates of the contributions of screening mammography and adjuvant treatment to the reduction of breast cancer mortality in the United States from 1975 to 2000 [68]. Seven independent statistical models were developed and yielded similar qualitative conclusions, namely that “the decline in mortality rate can be explained by a combination of screening and therapy and not by either one alone”. The proportion of the total reduction in the rate of death from breast cancer attributed to screening varied in the seven models from 28% to 65% (median 46%). On the other hand, the contribution of systemic therapy including chemotherapy and tamoxifen varied from 35% to 72% (median 54%): differences in these estimates reflect the mutual interaction between the two interventions [68].

Saadatmand et al. [69] investigated – in a large population-based cohort study – whether tumour stage at diagnosis still influences survival in the context of the current therapeutic approaches. This prospective nationwide population-based study was conducted in the Netherlands and included 173,797 women diagnosed with breast cancer, with two cohorts being identified according to the year of breast cancer diagnosis. A total of 80,228 patients were diagnosed with breast cancer from 1999 to 2005, while 93,569 from 2006 to 2012: in this second period – following national guidelines – systemic therapy was more widely used. At univariate and multivariate analyses, tumour stage and nodal status significantly influenced overall and relative survival in both cohorts. Relative survival rates ranged from almost 100% in both cohorts for in situ tumours to 57% and 59% for T4 tumours in the older and more recent cohort, respectively. At multivariate analysis, breast-conserving treatment (more frequently pursued in the 2006–2012 cohort) resulted in a significant survival benefit compared with mastectomy, whereas lymph node dissection (less frequently pursued) was associated with a significantly worse overall survival. The wider use of chemotherapy in the 2006–2012 cohort conferred a hazard ratio for death of 0.86 (95% CI 0.80–0.92). These large-scale results clearly demonstrate that while the use of chemotherapy may impact on survival, tumour size at diagnosis still matters. Authors concluded that “in the current era of effective systemic therapy, diagnosis of breast cancer at an early stage remains vital” [69]. Moreover, considering United States women diagnosed with breast cancer at age 60–74, summaries of the risk of breast cancer recurrence showed a 5-year recurrence rate of 2.5%, 9.6% and 34.5% for stages I, II and III HR-positive breast cancers, and a 5-year recurrence rate of 6.5%, 20.2% and 48.5% for stages I, II and III HR-negative breast cancers [24].

Another study from the United States [6] also assessed to what extent digital mammography screening and novel systemic therapies contributed to the improvement in breast cancer mortality in different disease subtypes from 2000 to 2012: its results are summarised in Table 1. Complex simulation models from the Cancer Intervention and Surveillance Network projecting breast cancer mortality trends for women aged 30 to 79 years estimated a 12% difference (model range, 10–16%) in the overall disease-specific mortality reduction between 2000 (37%, model range, 27–42%) and 2012 (49%, model range, 39–58%). The relative contribution to the decrease in overall breast cancer mortality in 2012 was 37% (model range, 26–51%) for screening and 63% (model range, 49–74%) for treatment. Of the 37% mortality reduction associated with screening in 2012, 33% (model range, 29–48%) was associated with screening advances before 2000 and 4% (model range, 1–8%) after 2000 (the shift from screen-film to digital mammography was relatively less relevant than previous improvements when considering the conspicuous advancements in therapeutic options). Similarly, of the 63% mortality reduction associated with treatments in 2012, 32% was associated with chemotherapy, 27% with hormone therapy and 4% with trastuzumab. Of the 31% mortality reduction (model range, 23–37%) associated with chemotherapy, 9% (model range, 7–14%) was associated with chemotherapy advances after 2000 (largely taxanes). Of the 27% mortality reduction (model range, 18–36%) associated with hormone therapy, 7% (model range, 2–12%) was associated with advances in hormone therapy after 2000 (largely from AIs) [6].

**Table 1:** Association of screening and treatment with breast cancer mortality in US women from 2000 to 2012

	Mortality reduction compared to 1975 (%)			Contribution to the difference in mortality reduction in 2012 versus 2000 (%)			
	In 2000^a^	In 2012^b^	Difference	Screening advances	Chemotherapy advances	Hormone therapy advances	Trastuzumab
Overall	37	49	12	17	38	29	15
ER+/HER2−	39	51	12	19	39	42	0
ER+/HER2+	39	58	19	12	22	25	41
ER−/HER2+	29	45	16	11	32	0	57
ER−/HER2−	29	37	8	22	78	0	0

^a^Relative to the estimated baseline rate of 64 deaths (model range, 56–73) per 100,000 women in 2000; ^b^Relative to the estimated baseline rate of 63 deaths (model range, 54–73) per 100,000 women in 2012. *ER* oestrogen receptor, *HER2* human epidermal growth factor receptor 2. Source: Plevritis et al. [6]

Subtype analyses also demonstrated significant variations in the relative contribution of screening and treatment to the mortality reduction in different tumour molecular subtypes. The largest benefit of screening (48%, model range, 38–57%) was found in the triple-negative tumour breast cancer cohort, while the largest benefit of treatment (69%, model range, 59–77%) was documented in luminal tumours. According to this model-based analysis, both screening and treatment contributed and still contribute to the improvement in breast cancer mortality, with progressively greater contributions of therapeutic advances in the last decades [6].

**Fig. 2:**
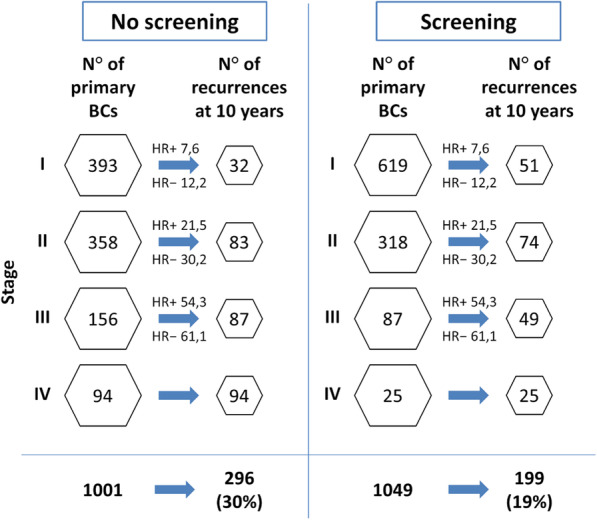
Effect of screening and chemotherapy on breast cancer recurrences among women aged 60 to 74 years, diagnosed with breast cancer between 1992 and 1999. Stage distribution was derived from Puliti et al. [21] considering attenders and non-attenders to screening mammography; 5% overdiagnosis attributed to screening mammography is taken into account. HR+ and HR− recurrences are back-calculated from Mariotto et al. [24]. The worst scenario reflects the absence of mammography screening and the use of chemotherapy alone. BCs, breast cancers; HR+, hormone receptor-positive breast cancers; HR−, hormone receptor-negative breast cancers

**Fig. 3:**
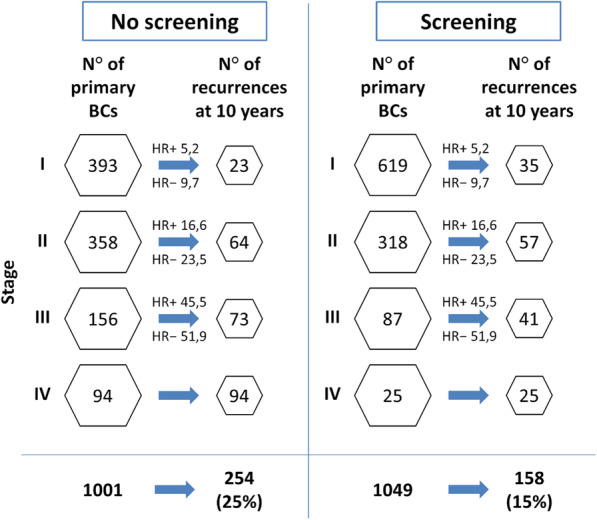
Effect of screening and novel systemic treatments on breast cancer recurrences among women aged 60 to 74 years, diagnosed with breast cancer between 2000 and 2013. Stage distribution was derived from Puliti et al. [21] considering attenders and non-attenders to screening mammography; a 5% overdiagnosis attributed to screening mammography was taken into account. HR+ and HR− recurrences are back-calculated from Mariotto et al. [24]. The most favourable scenario arises from the use of mammography screening associated with novel systemic treatments. BCs, breast cancers; HR+, hormone receptor-positive breast cancers; HR−, hormone receptor-negative breast cancers

The relative contribution associated with mammography screening and advances in systemic therapies to the reduction in the recurrence rate is simulated in Figs. 2 and 3. Here, four scenarios combine variably the contribution of screening mammography and chemotherapy or novel systemic treatments (i.e. AIs and targeted agents). In this simulation, stage distribution was derived from Puliti et al. [21], considering attenders and non-attenders to mammography screening and adjusted for self-selection. A 5% overdiagnosis attributed to population screening has been considered when it was included in the scenario. Ten-year disease-specific survival for HR-positive and HR-negative cancers was retrieved from Mariotto et al. [24], based on women aged 60–74 years treated in the 1992–1999 period or in the 2000–2013 period, when more advanced therapies were available. This simulation shows how combining screening mammography and novel systemic agents represent the most favourable scenario with the lowest number of recurrences, also detailing how the recurrence rate at 10 years would be 30% for chemotherapy without screening and 19% for chemotherapy with screening, 25% for novel treatments without screening and 15% for novel treatments with screening.

Two important aspects should however be considered in the analysis of the evolution of screening and systemic therapies. Despite the widespread use of screening mammography, the introduction of digital techniques represents the most important technical advancement: of note, tomosynthesis has become the technique of choice for symptomatic women and those who are recalled for further assessment after a screening mammogram [13]. Conversely, the evidence in favour of its use as a first-level screening tool is still not sufficient in terms of reduction of interval cancer rate (possibly due to an underlying rate of overdiagnosis [70–73]) and European guidelines [13] have advised against its use for population-based screening programs. On the other hand, increasingly effective novel systemic treatment options are being introduced as a standard of care and gene expression profiling is progressively enabling better decision-making.

This is once again a turning point. In case of further outcome improvements, most of the contribution would then be attributable to better systemic treatments. Nevertheless, MRI [74] and other novel imaging modalities, such as contrast-enhanced mammography [75–77] could come to be considered screening tools in selected populations, their contribution being also promising. Furthermore, a recent survey among members of the European Society of Radiology [78] showed that there are high expectations on the use of artificial intelligence [79, 80]. Dedicated algorithms will focus on personalised risk prediction and prognosis [81] and machine/deep learning software has already shown high performance in interpreting screening mammography. Finally, the availability of molecular analyses on liquid biopsy could represent another promising option [82, 83].

However, further widespread benefits in breast cancer outcomes are expected to become ever slimmer in the context of the efficacy of the current standard treatments: research efforts will therefore need to focus on the role of precision medicine. Once outcomes are maximal in the overall breast cancer patient population, improvements might indeed be sought in selected populations and especially in those with poor prognosis.

## Conclusions

The increasing use of screening mammography and improvements in systemic treatments have substantially reduced breast cancer mortality over the last two decades. However, defining their relative contribution to improving outcomes remains a challenging task. Early detection is crucial if followed by effective treatments. Nonetheless, treatments are still less effective in the case of advanced-stage disease. Prevention and early diagnosis contributed to almost half of the reduction in breast cancer mortality, whereas the rest is due to advances in breast cancer treatment, whose role and contribution has become predominant since the early 2000s. In the era of precision medicine, early detection remains crucial and a delay in breast cancer diagnosis, with a tumour detected at an advanced stage, can substantially increase mortality. Screening mammography and systemic anticancer treatment are synergistic in improving breast cancer prognosis.

## Data Availability

Not applicable.

## Abbreviations

AIs: Aromatase inhibitors
AUC: Area under the curve
CI: Confidence interval
CMF: Cyclophosphamide, methotrexate, and 5-fluorouracil
HER2: Human epidermal growth factor receptor 2
HR: Hormone receptor
